# Complete mitogenome of the high ethanol production fungus *Fusarium oxysporum* Mh2-2

**DOI:** 10.1080/23802359.2017.1398601

**Published:** 2017-11-20

**Authors:** Lu Zeng, Chichuan Liu, Runmao Lin, Xincong Kang, Bingyan Xie, Xingyao Xiong

**Affiliations:** a Horticulture and Landscape College, Hunan Agricultural University, Changsha, Hunan, China;; b Institute of Vegetables and Flowers, Chinese Academy of Agricultural Sciences, Beijing, China;; c College of Life Sciences, Beijing Normal University, Beijing, China

**Keywords:** Fusarium, plant infection, ethanol production, mitochondrial genome

## Abstract

*Fusarium* spp. are significantly important plant pathogens, and some of them are ethanol-producing strains. During infection and/or ethanol production, *Fusarium* requires a plenty of energy that is mainly provided by mitochondria. Here we report the first mitogenome from a selected *Fusarium oxysporum* strain mh2-2 that produces ethanol from glucose and xylose. The size of this mitogenome, 46 kb, is different from the size of any reported *Fusarium* mitogenome. Our results provide insight into the functions and evolution of mitochondrial genes and genomes.

*Fusarium* species include plant pathogens, opportunistic pathogens of humans and other animals, and non-pathogenic strains (Paulitz and Belanger 2001; Pietro et al. 2003; Ma et al. 2010). And some of *Fusarium* strains have the ability to degrade lignocellulose and consume pentose (de Almeida et al. 2013; Anasontzis and Christakopoulos 2014), serving as a renewable energy resource and making them a focus of scientific research.

Here we report the first mitogenome from a selected *Fusarium oxysporum* strain mh2-2 (FoxM) that produces ethanol from glucose and xylose. The fungus was originally isolated from the root of cotton in Urumqi, Xin Jiang, China (43:46N, 87:36E) and confirmed using ITS sequences. This isolate was deposited into the Guangdong Microbial Culture Collection Center (GDMCC 3.632). We find that the complete mitogenome of FoxM is 46 kb (GenBank accession no. MF155191) and it encodes 18 protein-coding genes, including 15 typical genes (three cytochrome c oxidase subunits, *cox1-3*; one apocytochrome b, *cob*; three ATP synthase subunits, *atp6* and *atp8*-*9*; seven subunits of NADH dehydrogenase, *nad1-6* and *nad4L*; one ribosomal protein, *rps3*) that are commonly found in fungal mitogenomes in Hypocreales (Lin et al. 2015), and three non-typical genes include one LAGLIDADG endonuclease (mh2-na2) and two hypothetical proteins. Additionally, the FoxM mitochondrial genome encodes 26 tRNAs and two rRNAs (*rns* and *rnl*). Compared with other three, *F. oxysporum* mitochondrial genomes have been reported (Cunnington 2007; Pantou et al. 2008; Brankovics et al. 2016), however, their sizes are quite different, ranging from 33 kb (FoxV) (Cunnington 2007) to 49 kb (FoxCR4) (Brankovics et al. 2016), in all four *F. oxysporum* mitochondrial genomes, both *rnl* and *nad5* contain one intron, and these introns encode *rps3* and mh2-na2 homologues, respectively. Compared with the other three *F. oxysporum* strains, the FoxCR4 *atp6* and *cob* genes have one and two more introns, respectively.

Moreover, we determined the phylogenetic relationships between *Nectria cinnabarina* (Wang et al. 2016), *Verticillium dahliae* (Pantou et al. 2008) and 10 *Fusarium* mitochondrial genomes based on the sequences of 14 typical protein-coding genes (*cox1-3*, *cob*, *nad1-6*, *nad4L*, *atp6*, and *atp8-9*). There were four major clades: clade A includes four *F. oxysporum* strains, clade B includes *F. circinatum* (31 proteins) and *F. verticillioides* (21 proteins) (Al-Reedy et al. 2012; Fourie et al. 2013), clade C contains *F. culmorum* (60 proteins), *F. gerlachii* (53 proteins), and *F. graminearum* (50 proteins) (Cuomo et al. 2007; Kulik et al. 2016a, 2016b), and clade D contains *F. solani* (30 proteins) (Al-Reedy et al. 2012) (Figure 1). All 10 *Fusarium* mitogenomes encode 15 typical protein-coding genes, but they encode different numbers of LAGLIDADG endonucleases, GIY-YIG endonucleases, and hypothetical proteins. The difference between phylogenetic clusters and gene numbers indicates significant differences in gene gains and losses in *Fusarium* mitogenomes during evolution. For example, compared with *F. solani*, strains in clade C gain more introns and endonucleases, *F. verticillioides* in clade B and four *F. oxysporum* strains in clade A lose many introns and endonucleases, while *F. circinatum* in clade B has similar numbers of introns and endonucleases. This study reports the complete mitogenome of ethanol production fungus FoxM for the first time and provides valuable information for further exploration of mitochondrial evolution.

**Figure 1.**
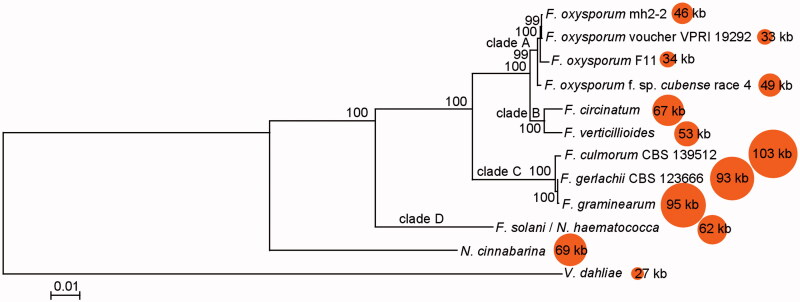
Phylogenetic relationship between *Fusarium oxysporum* mh2-2 and other 11 fungal mitogenomes. The maximum-likelihood tree was generated by MEGA v6.06 (Tamura et al. 2013) based on 14 concatenated protein-coding genes (*nad1-6*, *nad4L*, *atp6*, *atp8-9*, *cob,* and *cox1-3*). *Verticillium dahliae* was used as outgroup. The model for phylogenetic analysis was GTR + G + I with bootstrap value of 1000.
